# Optimizing Music Learning: Exploring How Blocked and Interleaved Practice Schedules Affect Advanced Performance

**DOI:** 10.3389/fpsyg.2016.01251

**Published:** 2016-08-18

**Authors:** Christine E. Carter, Jessica A. Grahn

**Affiliations:** ^1^School of Music, Memorial University of Newfoundland, St. John’s, NLCanada; ^2^Department of Psychology, Western University, London, ONCanada

**Keywords:** practice, performance, learning, training, contextual interference effect

## Abstract

Repetition is the most commonly used practice strategy by musicians. Although blocks of repetition continue to be suggested in the pedagogical literature, work in the field of cognitive psychology suggests that repeated events receive less processing, thereby reducing the potential for long-term learning. Motor skill learning and sport psychology research offer an alternative. Instead of using a blocked practice schedule, with practice completed on one task before moving on to the next task, an interleaved schedule can be used, in which practice is frequently alternated between tasks. This frequent alternation involves more effortful processing, resulting in increased long-term learning. The finding that practicing in an interleaved schedule leads to better retention than practicing in a blocked schedule has been labeled the “contextual interference effect.” While the effect has been observed across a wide variety of fields, few studies have researched this phenomenon in a music-learning context, despite the broad potential for application to music practice. This study compared the effects of blocked and interleaved practice schedules on advanced clarinet performance in an ecologically valid context. Ten clarinetists were given one concerto exposition and one technical excerpt to practice in a blocked schedule (12 min per piece) and a second concerto exposition and technical excerpt to practice in an interleaved schedule (3 min per piece, alternating until a total of 12 min of practice were completed on each piece). Participants sight-read the four pieces prior to practice and performed them at the end of practice and again one day later. The sight-reading and two performance run-throughs of each piece were recorded and given to three professional clarinetists to rate using a percentage scale. Overall, whenever there was a ratings difference between the conditions, pieces practiced in the interleaved schedule were rated better than those in the blocked schedule, although results varied across raters. Participant questionnaires also revealed that the interleaved practice schedule had positive effects on factors such as goal setting, focus, and mistake identification. Taken together, these results suggest that an interleaved practice schedule may be a more effective practice strategy than continuous repetition in a music-learning context.

## Introduction

Observations of student music practice consistently show that repetition is the most employed practice technique (Barry, 1992, 2007; Rohwer and Polk, 2006). This is supported by the pedagogical literature, in which musicians are frequently encouraged to repeat challenging passages multiple times in a row (e.g., Hadcock, 1999; West, 2003; Fischer, 2004). In psychology, this type of repetition-focused practice is called *blocked* practice, whereby practice on one task is completed before moving onto a second task (Williams, 2006). Blocked practice seems intuitive. As a task is continually repeated, it starts to feel more fluent, and this increased fluency leads learners to think that blocked repetition is effective. It turns out, however, that immediate fluency and perceived gains during training are not good indicators of long-term learning (Bjork, 1999). Musicians often return to the practice room only to find that the results of the previous day’s practice were not retained (Stambaugh, 2011b).

An alternative to blocked practice is *interleaved* practice, which involves practicing multiple tasks concurrently by alternating between them. A large body of research has found that, while interleaved practice may impede performance during training compared to blocked practice, it increases long-term learning (for a review, see Magill and Hall, 1990). This finding, called the *contextual interference effect*, was initially documented in studies of word-pair learning (Battig, 1966), and has since become a major focus in research on motor skills. Shea and Morgan (1979) found that when participants practiced a set of barrier knock-down patterns in an interleaved schedule, they had faster response times at 10-min and 10-day delayed retention testing than those who practiced the tasks in a blocked schedule. This seminal study launched a series of experiments examining practice schedules in motor skill learning (e.g., Maslovat et al., 2004) and sport psychology. Hall et al. (1994), for example, tested the contextual interference effect in baseball. Elite players were offered 6 weeks of additional bi-weekly batting practice, either in a blocked, or interleaved schedule. Participants in the blocked schedule practiced hitting 15 fastballs in a row, followed by 15 curve-balls, followed by 15 change-up pitches. Participants in the interleaved group also practiced hitting 15 of each of the 3 pitch types, but in a random order. When compared with the participants’ level at the initial pre-test, the interleaved group hit an additional 56.7% of the pitches, the blocked group 24.8%, and the control group, who received no additional practice, 6.2%. The improvement for the interleaved group was almost twice that of the blocked group, even though both received an identical number of practice pitches. The superiority of interleaved over blocked practice has also been found for badminton (Wrisberg, 1991), golf (Guadagnoli et al., 1999; Porter et al., 2007), and snowboarding (Smith, 2002), among other sports.

There are two predominant hypotheses that explain the contextual interference effect, each of which has received empirical support. The *elaborative-processing hypothesis* suggests that different tasks being practiced together reside simultaneously in working memory. If multiple items are held in working memory at the same time, there is an opportunity to compare and contrast the different items, leading to a more elaborate and distinctive encoding for each item (Lee and Simon, 2004). The *forgetting-reconstruction hypothesis* suggests a very different process. Switching from one task to another may induce forgetting of the previous task’s action plan. It is the reconstruction of action plans upon return to prior tasks that leads to a stronger memory representation (Magill and Hall, 1990). Despite the apparent mutual exclusivity of these two theories, it is plausible that they could work in tandem. Lee and Simon (2004, p. 36) suggest that while constructing an action plan, “one could make comparisons and contrasts with the previous action, whilst essentially replacing it as the ‘loaded’ response.” Whether a combination of the theories is possible or not, it is clear that both explanations hold that interleaving practice on different tasks increases effortful cognitive processing, and this in turn benefits retention.

Despite the extensive motor skill practice required for developing musical expertise, research on the contextual interference effect in music is limited. Stambaugh (2009) found that fifth and sixth-grade beginner clarinet students who practiced 3 simple 7-note musical stimuli in an interleaved schedule were able to play faster at retention than those in the blocked schedule. Other studies, however, were less conclusive. Seventh-grade clarinetists and saxophonists who practiced 8-measure musical stimuli in blocked, interleaved, and a hybrid schedule showed no effect of schedule for technical accuracy, and, curiously, both the blocked and interleaved schedule groups performed more musically than the hybrid group at retention (Stambaugh and Demorest, 2010). A subsequent study involving university-level participants verified the contextual interference effect for woodwind players, but not brass players (Stambaugh, 2011a).

The present study builds upon the existing contextual interference research in music by varying a number of parameters, including the length and type of music practiced, the length and structure of the practice sessions, the type of analytical designed used, and the approach to retention assessment. All of these parameters were varied in order to create a more ecologically valid practice environment, more closely resembling the type of work undertaken by musicians. Whereas previous studies used short musical stimuli ranging from 7 notes (Stambaugh, 2009, 2011a) to 8 measures (Stambaugh and Demorest, 2010), the current study used longer musical stimuli taken from preexisting musical sources (e.g., concerto expositions and technical exercises). Prior studies also used relatively short practice sessions, with a maximum of 6 min spent on each stimulus. The current study doubled the practice time devoted to each musical stimulus to 12 min. To maximize statistical power, the present experiment also used a within-subjects design, with each participant experiencing both the blocked and interleaved conditions. Finally, prior studies assessed specific markers, such as speed or pitch accuracy, to determine the effect of different practice schedules. The current study instead had three professional musicians assess overall performance improvement, as would take place in a typical music jury or competition.

Thus, the purpose of this study was to examine the effects of interleaved and blocked practice schedules on advanced clarinet performance in an ecologically valid practice environment. Does interleaved practice benefit advanced clarinet performance when using authentic musical stimuli, longer practice sessions, and real-world outcome assessments?

## Materials and Methods

### Participants

Following approval by Western University’s Psychology Research Ethics Board, clarinetists were recruited from Western and the surrounding London (Ontario) area by poster and email advertisement. In order to participate, individuals had to be at least 18 years of age and had to have played the clarinet for a minimum of 4 years, with a minimum of 2 years of private study on the instrument. Additionally, participants were required to play the clarinet at least 8 h per week in order to be included in the experiment. The ten participants included six men and four women, who ranged in age from 19 years old to 55. Nine of the 10 participants were between the ages of 19 and 22. On average, the participants had played the clarinet for 8.75 years, had taken private lessons on the clarinet for 6.35 years, and played the clarinet 18.6 h per week. Participants also ranked their ability on a scale of 1 to 10 with “1” representing a clarinetist who had just had their first lesson and “10” representing a top professional clarinetist. The rankings ranged from 5 to 7.5 and averaged 6.5. Demographic details for each participant are listed in **Table 1** below. Participants took part in a one-and-a-half hour practice session on one day followed by a 30-min follow-up session the following day. A nominal honorarium of $10 (CAD) for the first day and $5 for the second day was given to all participants for completing the study.

**Table 1:** Participant demographics.

Participant	Age	Years of clarinet playing experience	Years of private clarinet lessons	Average hours playing per week	Hours played last week	Sex	Self-rating
1	22	5.5	5.5	8.5	21	M	6
2	21	7	7	22.5	20	F	7.5
3	19	11	11	25	28	M	7
4	20	8	5	25	25	M	7
5	22	11	5	30	20	F	7
6	22	7	6	13	14	F	6.5
7	21	9	7	11	10	F	6.5
8	55	9	7	7	9	F	5
9	20	10	5	30	40	F	6.5
10	22	10	5	14	11	M	6
Mode	22	7, 9, 10, 11	5	25, 30	20	F	6.5, 7
Median	21.5	9	5.75	18.25	20	NA	6.5
Average	24.4	8.75	6.35	18.6	19.8	NA	6.5
Standard deviation	10.25	1.75	1.76	8.39	9.08	NA	0.67

### Stimuli

Musical stimuli consisted of two comparable eighteenth century concerto expositions by Karl Stamitz, including one in F major and one in E-flat major (Stamitz, 1956, 1970) and technical exercises numbered 6 and 19 from Jean-Xavier Lefèvre’s, *Méthode de clarinette* (Lefèvre, 1967). The concerto expositions were chosen to provide authentic musical scores, containing a variety of technical and musical elements. The first concerto exposition was 52 measures and the second was 49 measures. Both were entered into Finale to create new editions that were equivalent in size and format. The technical studies were chosen to provide repertoire with sustained technical demands that would allow for significant improvement over practice. Both technical studies were 16 measures long. All selected pieces are rarely performed, ensuring that participants would not have received previous training on the repertoire. This novelty was confirmed with participants in a questionnaire following the study. The four musical stimuli can be viewed in the Supplementary Material.

### Procedure

Participants were provided with a letter of information upon arrival and gave written informed consent prior to beginning the study. In the first testing session, each participant completed two consecutive practice sessions, one in a blocked schedule (low contextual interference condition), and one in an interleaved schedule (high contextual interference condition). In the blocked schedule condition, participants sight-read one of the concerto expositions, and then practiced it for 12 min, followed by sight-reading and practicing one of the technical studies for 12 min. In the interleaved schedule condition, participants sight-read the remaining concerto exposition and technical study and then alternately practiced them, switching between pieces every 3 min. The practice breakdown for each condition can be seen in **Table 2**.

**Table 2:** Blocked and interleaved practice schedule breakdown.

Blocked schedule (minutes)	Interleaved schedule (minutes)
3 (approximately) Read Concerto Exposition 1	3 (approximately) Read Concerto Exposition 2
12 Practice Concerto Exposition 1	1 (approximately) Read Technical Study 2
1 (approximately) Read Technical	
Study 1	3 Practice Concerto Exposition 2
12 Practice Technical study 1	3 Practice Technical Study 2
=Approximately 28 min	3 Practice Concerto Exposition 2
	3 Practice Technical Study 2
	3 Practice Concerto Exposition 2
	3 Practice Technical Study 2
	3 Practice Concerto Exposition 2
	3 Practice Technical Study 2
	=Approximately 28 min

Apart from adhering to the instructed timing, participants were told to practice each excerpt as though they were in their own practice room. Immediately following practice, participants performed all of their excerpts in an acquisition trial, following instructions to play as “accurately and musically as possible.” The run-through at the end of Day 1 provided the measure of immediate practice retention, without an intervening delay. The total time for the first session did not exceed one-and-a-half hours. One day later, participants returned to the lab to perform their excerpts again in a retention trial. This second session did not exceed 30 min. Participants were allowed to warm up and then were asked to play each of the excerpts from the day before. This provided the measure of retention after a delay. All practice sessions and acquisition/retention trials were recorded using a portable Zoom recording device.

Following the run-through, participants completed a musical background questionnaire to establish demographic information. They were also asked questions about the blocked and interleaved schedules in the study, including which schedule they preferred, which schedule they found the most useful, and which schedule was closest to the one used in their daily practice. Additional comments on their experience in the two practice conditions were noted. Upon their departure from the lab, participants were given a debriefing form with further information about the study.

### Design

The study followed a within-subjects design, in which each participant experienced both the blocked and interleaved conditions, in order to control for individual differences between participants. The order of conditions was counterbalanced, as were the two musical stimuli in each condition. In other words, half of the participants started with the blocked condition and half started with the interleaved condition. Additionally, some participants practiced the first concerto exposition in the blocked condition and the second concerto exposition in the interleaved condition, while others practiced the first concerto exposition in the interleaved condition and the second concerto exposition in the blocked condition. The same counterbalancing was applied to the two technical studies to ensure that any results were not due to the pieces themselves, but rather to the practice conditions in question.

The quality of the acquisition (end of Day 1) and retention (Day 2) performance trials was determined by blind ratings of the trial recordings. Recording raters consisted of three professional clarinetists, paid a $100 (CAD) honorarium for their time. Raters 1 and 3 were retired university clarinet professors and Rater 2 was a principal clarinetist in a Canadian orchestra. Raters were given four CDs with each participant’s audio recordings grouped by piece. The first recording for each piece was always the sight-reading run-through. This was followed by the acquisition and retention trials, however the order of these was counterbalanced from piece to piece and deliberately not indicated on the CDs. Raters were instructed to compare the second and third recordings of each piece (acquisition and retention) to the sight-reading performance before giving a percentage rating for each trial. **Table 3** contains the complete information given to raters.

**Table 3:** Information given to raters.

**For your information:** There are a total of 10 participants. Each participant plays 4 pieces (the 2 technical exercises and 2 pieces). Each piece is recorded 3 times. The recordings are grouped in 3 by piece. The first recording in each group is a sight-reading run-through. The next two occurred after sight-reading, but are in no particular temporal or condition order.
**Focus:** We are most interested in the difference between the sight-reading performance and the subsequent two performances.
**Rater Instructions:** Please listen to tracks in their entirety. The two technical excerpts are quite short (less than 1 min). The two concerto expositions are just over 2 min. It is important to listen to the entire expositions, as larger technical passages end both of them.
Listen to Track 1 on CD 1. This is an example of a performance that would receive a lower score among the examples (e.g., 55%).
Listen to Track 2 on CD 1. This is an example of a performance that would receive a higher score among the examples (e.g., 95%).
Keep this range in mind when you are assigning scores. Try to utilize a broad range of scores (e.g., 50–100) rather than a narrow range of scores (e.g., 65–75).
Assign scores based on the overall performance (e.g., combination of accuracy, fluidity of technique, musicality), rather than on only one specific characteristic.
Listen to the remaining tracks.
Rate the sight-reading performance for each piece first. Then rate the two interleavedly ordered performances that follow, directly comparing how each relates to the sight-reading performance.
E.g., If you hear a mediocre sight-reading performance followed by a much improved performance, followed by a minimally improved performance (always comparing back to the sight-reading version), the scores for a particular piece might look like this: 65, 83, 72.
The four CDs are between 35 min and 1 h in length. You do not need to rate all of these in one sitting! Feel free to split up your listening/rating, as long as you always finish any set of 3 performances (sight-reading and subsequent 2 performances of a particular piece) before stopping.

Non-parametric statistics were used to analyze the data because of the small sample size. Specifically, data were analyzed with paired sample sign tests and paired sample Wilcoxon signed rank tests, which are non-parametric equivalents of the paired samples *t*-test. The tests were used to statistically compare the amount of rating improvement from sight-reading to end of day 1 and sight-reading to end of day 2. In contrast to the Wilcoxon test, the sign test does not take into account the magnitude of the improvement, only whether there was improvement, no change, or a decline. The sign test may sometimes be less powerful than the Wilcoxon sign test for this reason (Lowry, 2013).

## Results

To determine if the type of practice schedule (blocked or interleaved) had an effect on improvement following practice, the Wilcoxon signed rank test and sign test were used to compare the raters’ blocked and interleaved percentage scores. First, the improvement from the initial sight-reading trial to the delayed retention test on day 2 was assessed to determine overall improvement. Second, the improvement from the end of day 1 to day 2 was assessed to indicate how much progress was retained from the end of practice on day 1 to the run-through on day 2.

**FIGURE 1:**
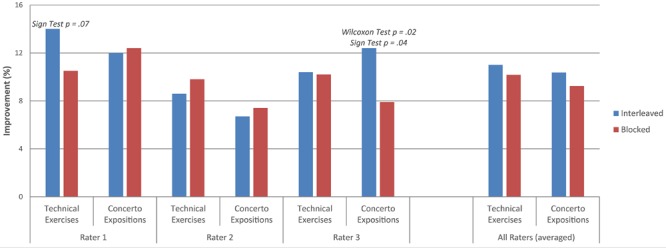
**Average overall improvement from sight-reading to day 2**.

Results varied across raters. For improvement from sight-reading to day 2, technical performance in the interleaved condition was rated as better than the blocked condition by rater 1 (sign test, marginally significant: *p* = 0.07, see **Figure 1**). Raters 2 and 3 did not show any significant differences in ratings for interleaved and blocked technical conditions. For concerto expositions, performance in the interleaved condition was rated as better than the blocked condition by rater 3 (Wilcoxon signed rank test, *p* = 0.02; sign test, *p* = 0.04). Raters 1 and 2 did not show any significant differences in concerto ratings for interleaved and blocked conditions. There were no other significant findings from sight-reading to day 2. The average overall improvement scores from sight-reading to day 2 for each rater, as well as all three raters combined, are presented in **Figure 1**.

**FIGURE 2:**
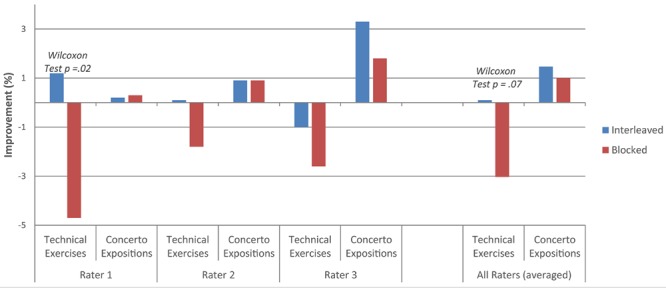
**Average improvement from the end of day 1 to day 2**.

For improvement from the end of day 1 (after practice was completed) to day 2, technical performance in the interleaved condition was rated as better than the blocked condition by rater 1 (Wilcoxon signed rank test, *p* = 0.02, see **Figure 2**), and by all three raters combined (Wilcoxon signed rank test, marginally significant: *p* = 0.07). There were no other significant findings from the end of day 1 to day 2. The average improvement scores from the end of practice on day 1 to day 2 for each rater and all three raters combined are presented in **Figure 2**. Please note that a negative improvement score indicates that performance worsened from the end of day 1 to day 2.

Beyond the significant and marginally significant results discussed above, the interleaved condition was always superior to the blocked condition when averaged across raters, but not enough to reach significance using the Wilcoxon signed rank test.

In order to quantify variability across raters, Pearson correlations were calculated for each pair of raters (raters 1 and 2, raters 1 and 3, and raters 2 and 3). These correlations were then averaged across pairs producing the overall inter-rater reliability values included in **Table 4** below.

**Table 4:** Pearson correlations (r) averaged across rater pairs.

Condition	Overall improvement from sight-reading to day 2	Improvement from end of day 1 to day 2
Technical exercises interleaved	*r* = 0.59	*r* = 0.28
Technical exercises blocked	*r* = 0.51	*r* = 0.39
Concerto expositions interleaved	*r* = 0.22	*r* = 0.49
Concerto expositions blocked	*r* = 0.22	*r* = 0.02

The questionnaire data revealed that, while all ten participants used a blocked schedule in their daily practice, six found the interleaved schedule in the study more useful than the blocked schedule in the study (three found the blocked schedule more useful and one found them equal). In addition, four preferred the interleaved schedule to the blocked schedule (six preferred the blocked schedule). The data for each participant is below in **Table 5** and additional comments regarding participants’ experience in each practice schedule follow in **Table 6**. Of the eight participants who commented on the practice styles, all eight made favorable comments about the effect of interleaved practice on factors such as goal setting, focus, and mistake identification.

**Table 5:** Typical, most useful, and preferred participant practice schedules.

Participant	Which schedule type is closest to the way your normally practice?	Which schedule type did you find most useful?	Which schedule type (blocked or interleaved) did you prefer?
1	Blocked	Blocked	Blocked
2	Blocked	Interleaved	Blocked
3	Blocked	Interleaved	Interleaved
4	Blocked	Interleaved	Interleaved
5	Blocked	Interleaved	Interleaved
6	Blocked	Interleaved	Blocked
7	Blocked	Blocked	Blocked
8	Blocked	(Same)	Blocked
9	Blocked	Interleaved	Interleaved
10	Blocked	Blocked	Blocked
Total blocked:	10	3	6
Total interleaved:	0	6	4
Total tied:	0	1	0

**Table 6:** Participant comments regarding the two practice schedules.

Participant	Comments
1	NA
2	“Although the blocked practice is more representative of my practice style, I did feel that the interleaved practice made me more goal-oriented since there were only 3 min spurts.”
3	“For the interleaved practice, there were shorter periods of time, so I had to focus in on what I was doing. In the longer blocks, it was easier for my mind to wander.”
4	“In the interleaved condition, everything was fresh when I would come back to the pieces, but when I had to perform them, I remembered them more. In the blocked condition, it felt like I was sight-reading again at the end of the day.”
5	“Blocked time is normally what I am used to. With the 3-min segments, I had to organize what I was going to work on much faster. During the blocked time, I didn’t organize my practice as quickly. I would still target what I didn’t like about it, but you can get lost in the longer segment.”
6	“I don’t usually practice in the interleaved schedule. Yesterday I was forced to switch between the pieces. Every time I would go back to a piece, I would have a new outlook and it seemed like a new practice. I thought that helped me identify the mistakes I made the previous time. Yesterday I felt that the pieces from the interleaved practicing improved at the end of the day. Some of the things I worked on yesterday in the interleaved condition carried over to today. In the interleaved condition, I felt like the time limit forced me to abandon the idea I was working on. I think it would have been better if I could finish what I’m working on and then move onto the next piece.”
7	“Having a pencil to mark in mistakes would have helped.”
8	“I didn’t feel like the two types of practice made a difference. But while I was doing the practice, the 3-min sessions were a bit more interesting. I might try that again sometime, because taking a break from it might be a good thing. Previously I experienced that if I took a break from something for over a week, it was better when I came back to it. Maybe on a shorter term, it would work the same way.”
9	“I was less frustrated going back and forth [interleaved], because if there is something I couldn’t play, switching to something else and then going back clears your mind. Knowing I had such a short period of time, I was more focused in the time that I did have.”
10	“The Concerto in F was the only one in concert F, so I missed a lot of F-sharps and played B-flats because I was used to the key signature of the other three pieces. Once I got into it, it wasn’t as bad. I felt much less comfortable in the interleaved schedule, but it did help me pace myself.”

## Discussion

The purpose of this study was to assess the generalizability of the contextual interference effect to an ecologically valid advanced music learning setting. Specifically, this experiment tested the effect of blocked and interleaved practice schedules on the learning of technical exercises and concerto expositions by ten advanced clarinetists. Analyses of overall improvement from sight-reading to day 2 revealed a marginally significant advantage for technical exercises practiced in the interleaved schedule (rater 1) as well as a significant advantage for concerto expositions practiced in the interleaved schedule (rater 3). Analyses of improvement from the end of day 1 to day 2 revealed a significant advantage for technical exercises practiced in the interleaved schedule (rater 1), which became marginally significant when averaged across all three raters. The interleaved schedule produced greater improvement than the blocked schedule when scores were averaged across raters, although not enough to reach statistical significance beyond the specific example listed above.

The statistically significant findings in favor of interleaved practice are consistent with Stambaugh’s (2009, 2011a) previous studies of beginning clarinetists and university woodwind players, in which players of both experience levels were able to play significantly faster following interleaved practice than blocked practice. The current results suggest that interleaved practice may also benefit woodwind players in real-world contexts, when practicing their assigned music and assessed for overall performance by professional musicians. The present study also produced a number of findings that did not reach statistical significance, consistent with other studies by Stambaugh and Demorest (2010), Stambaugh (2011a) with middle school students, in which interleaved practice did not benefit technical accuracy and with university brass players, in which there was no benefit of interleaving. The inconsistency in findings may in part be due to the challenge of studying the contextual interference effect in applied, highly variable settings. Such inconsistencies have also been seen in studies of applied sport psychology (Barreiros et al., 2007). It will be important to determine if these inconsistencies are due to study design considerations that can be further explored in the future.

The empirical results of this study, while modest in size, do suggest that an interleaved schedule may be a viable alternative to a blocked schedule in the practice room. In addition to potential gains in improvement over a blocked schedule, the interleaved schedule provides a more realistic performance context when considering demands in an audition or concert setting. While it has been established that musicians rely extensively on blocked repetition in the practice room, repeated attempts are not possible in a real-world performance; there is only one chance to start each piece in the concert hall. By continually switching between tasks, an interleaved schedule creates processing that is more likely to transfer, facilitating multiple opportunities to start the material anew, as is necessary in performance. Interleaved practice may also have implications for musicians’ health. Repetitive strain injury is observed in over 60% of musicians (Hoppmann and Patrone, 1989). Shifting away from overly repetitive practice structures may help reduce the prevalence of this disorder. Interleaving builds in physical variety, avoiding the constant repetitive movements necessary in blocked practice.

The loss in performance observed from day 1 to day 2 for technical exercises practiced in a blocked schedule highlights the fact that performance immediately following practice is an imperfect indicator of long-term learning. A practice strategy that creates the most improvement during practice may not be the best strategy for creating long-term improvement. It is therefore crucial to include a retention phase in any test of learning, “conducted after an interpolated interval that is long enough to ensure that any temporary effects of the independent variable have been dissipated” (Schmidt and Bjork, 1992, p. 208). Improvements from practice do not necessarily persist from one day to the next, and the amount of enduring improvement, the real measure of learning and the ultimate goal of practice, is affected by the type of processing involved during training.

In addition to the obvious importance of the durability of practice gains, the relationship between performance directly following practice and a subsequent delay might also affect performer motivation and confidence. If a performer’s practice improvements do not persist over even a short intervening interval, the performer may associate performance losses with their own inefficacy. Optimizing long-term learning over short-term gains may, therefore, have a positive impact on psychological factors involved in learning and performance.

Beyond the empirical findings discussed, additional information was provided by the post-experiment participant questionnaire. All ten participants stated that their typical practice structure most resembles the blocked schedule used in the study, lending further support to the well-documented use of continuous repetition in the practice room. Although no participants reported using an interleaved schedule in their daily practice, six found this schedule more useful than the blocked schedule. Eight of the ten participants also made favorable comments about the effect of the interleaved schedule on factors such as goal setting, focus, and mistake identification, all critical components of effective practice (Williams, 2006). Both the perceived usefulness of the interleaved schedule and related positive effects on performance-enhancing variables are promising regarding the potential implementation of the interleaved schedule as a regular practice tool.

It is worth noting that, while the majority of participants found the interleaved practice schedule more useful than the blocked schedule, the majority still preferred the blocked schedule. This supports previous findings that blocked practice is often favored over more challenging training conditions because of increased feelings of fluency after repetition. “Presenting the same item twice consecutively makes processing the second presentation seem highly fluent, providing a (misleading) impression of learning….” (Kornell and Bjork, 2008, p. 586). Interleaving, in contrast, decreases feelings of fluency during practice, and may lead learners to underestimate how much they will retain. In a study of artist style learning, for example, Kornell and Bjork (2008) found that 78% of participants said that blocked practice was equal to or better than interleaved practice, even though 78% of participants were more accurate following interleaved practice than blocked practice. Interestingly, participants made these judgments *after* the tests that clearly showed the superiority of interleaved practice. Feelings of fluency can have a powerful impact over judgments of learning, regardless of how much learning has taken place. This underlines the importance of considering metacognitive factors when studying learning, especially for practice techniques that introduce difficulties (albeit beneficial ones) for the learner.

### Recommendations for Future Research

The current study extended previous contextual interference research in music by using longer and more realistic stimuli, longer holistic practice sessions, and a within-subjects design to control for individual differences in performance ability. In addition to these participant-centered design changes, an ecologically valid rating system was used, with multiple expert raters giving overall performance percentages for pieces, as would be done in conservatory juries.

Given the small sample size of ten participants, the empirical and qualitative results discussed are promising. In order to establish the basis for a broad application of interleaved practice schedules in music, however, further research is necessary. Although this study’s attempt at ecologically valid practice and assessment is critical in terms of potential real-world applications, specific design modifications may allow larger effect sizes to be observed.

The first suggestion for future research is to conduct a longer study with multiple practice sessions distributed over a number of weeks. Due to the present study’s requirement that each participant practice two pieces in both the interleaved and blocked conditions, actual practice on each piece was limited to 12 min. Even so, this led to a demanding 90-min practice session when combined with the sight-reading and post-practice run-throughs. Shea et al. (1990, p. 147) indicate that contextual interference studies often use “relatively few acquisition trials,” and that the benefits of interleaved practice over blocked practice may not become apparent until later in practice. Increasing the overall time spent practicing each piece significantly beyond the 12-min intervals employed in this experiment may magnify the results discussed above.

Increasing the sample size should also be considered in future research. As the number of participants goes up, so does the statistical sensitivity to the effects of each condition. With a sample of only ten participants, large effects are needed to show significance, while subtler, but still important, changes may go unnoticed. It should be noted that the current sample size of ten participants required that the expert raters each listen to and rate 4 h of recordings, so any changes to sample size must also weigh requirements placed on raters. Varying the length of musical stimuli may help modulate this impact.

Although the holistic approach to rating is akin to real-world music assessment contexts, there is a subjective element to this type of evaluation. All three raters were given identical instructions, yet variability in their responses was evident. Pearson correlations, averaged across rater pairs, range from 0.02 to 0.59 for improvement scores. While these correlations are all positive, showing an inter-rater relationship in the right direction, they are not very consistent. This parallels Thompson and Williamon’s (2003) findings on real-world musical performance assessment, in which correlations between evaluator ratings were only moderate. Such variance highlights how unequable raters’ judgements can be, even when given the same rating scale and guidelines. Variability across raters may also obscure true beneficial effects present in the performances. While not practical in an educational context, using a larger number of evaluators may help reveal which patterns of performance improvement are consistently observed in future studies. Thompson and Williamon are also exploring whether analyzing individual rater differences may provide a useful method of improving reliability ratings: “If a number of self-assessment measures are included *within* the assessment procedure and completed by the evaluators themselves, these can then be used as covariates in subsequent statistical analyses, providing a means of controlling for biases.” Ultimately, supplementing real-world evaluation methods with more reliable metrics may provide the best balance between ecological validity and empirical rigor.

In addition to the modifications discussed above, future research could extend to other instrumental and non-instrumental groups and a diverse range of ability levels including beginners and professionals. Trying various divisions of time could also be explored for the interleaved condition, as well as the use of an increasing schedule of contextual interference, in which initial practice is repetitive and gradually becomes more interleaved as competence increases (Porter and Magill, 2010). Such forays may help determine in which settings and for which people specific practice schedules are most appropriate.

Finally, it would be worth expanding the current study’s focus on applied performance to other areas in music, such as ear training, theory, and history. Robust findings in a diverse range of fields, including mathematics, art, and handwriting skills, suggest that the contextual interference effect may have further widespread applications in a music-learning context and beyond (e.g., Ste-Marie et al., 2004; Kornell and Bjork, 2008; Taylor and Rohrer, 2010).

## Conclusion

Repetition, the most utilized technique by musicians in the practice room, continues to be endorsed by music pedagogues as the path to mastery. An article on effective practice techniques in *American String Teacher* states that repetition is necessary in order to make movements “more refined and automatic” and so that “less effort is expended” (Tatton, 1997, p. 56). This is well-intentioned advice; it seems logical that repetition would lead to better performance. The article goes on to suggest that, “thoughtless repetition, however, is boring and less than productive. Mental involvement is above necessary for successful practice and eventual mastery.”

Although it is recognized that thoughtless repetition is not effective, one often assumes “mental involvement” can be turned on at will, regardless of the amount of repetition. This may rely on the impression that after the initial attempt at a task subsequent repetitions are subject to the same amount of mental processing. Unfortunately, this is not the case. “It is incorrect to conclude that because an event is repeated the processing of that event is also repeated. Rather, repetition of an event can result in the solution being remembered without the necessity of engaging in the activities that would otherwise be required to obtain that solution” (Jacoby, 1978, p. 666).

If, for example, one solves the mathematical problem of 143 + 247 and determines that the answer is 390, trying to solve the problem again immediately after the first attempt will result in remembering the solution of 390, without the need to go through the initial arithmetical steps. Such repetitions will not lead to improved addition skills.

Consciously trying to engage mental involvement in a context that automatically turns off processing is counter-productive. Instead, practice techniques should be introduced that provide “desirable difficulties” for the learner, inherently requiring more effortful processing (Bjork, 1994). Increasing contextual interference through an interleaved practice schedule is one such technique.

The potential benefits of contextual interference in music practice depend on its successful implementation. Given the desire for immediate results in the practice room and instructional setting, there may be resistance to implementing a technique that slows performance gains in practice, even though the long-term effects are superior. “Rapid progress in the form of improved performance is reassuring to the learner, even though little learning may be taking place, whereas struggling and making errors are distressing, even though substantial learning may be taking place. Such a misreading of one’s progress…can lead trainees to prefer less effective training over more effective training” (Bjork, 1994, p. 194).

In order for contextual interference to be adopted, the repeat-until-perfect paradigm will need to be renounced in favor of a model that embraces challenge as a long-term performance enhancement strategy, despite limited instant gratification. This requires a perceptual shift by trainers and trainees alike.

## Author Contributions

All authors listed have made substantial, direct and intellectual contribution to the work, and approved it for publication.

## Conflict of Interest Statement

The authors declare that the research was conducted in the absence of any commercial or financial relationships that could be construed as a potential conflict of interest. The reviewer TC and the handling Editor declared their shared affiliation, and the handling Editor states that the process nevertheless met the standards of a fair and objective review.
