# Supplementary material for: Optimizing Music Learning: Exploring How Blocked and Interleaved Practice Schedules Affect Advanced Performance
Source: Front Psychol. 2016 Aug 18;7:1251. doi: 10.3389/fpsyg.2016.01251 (PMC4989027; doi:10.3389/fpsyg.2016.01251)

Supplementary Figure 1: Karl Stamitz, Clarinet Concerto in F Major: I, Exposition
© Universal Music Publishing Editio Musica Budapest (1970), Budapest, all rights reserved

Solo-Klarinette in B♭

Konzert in F-Dur

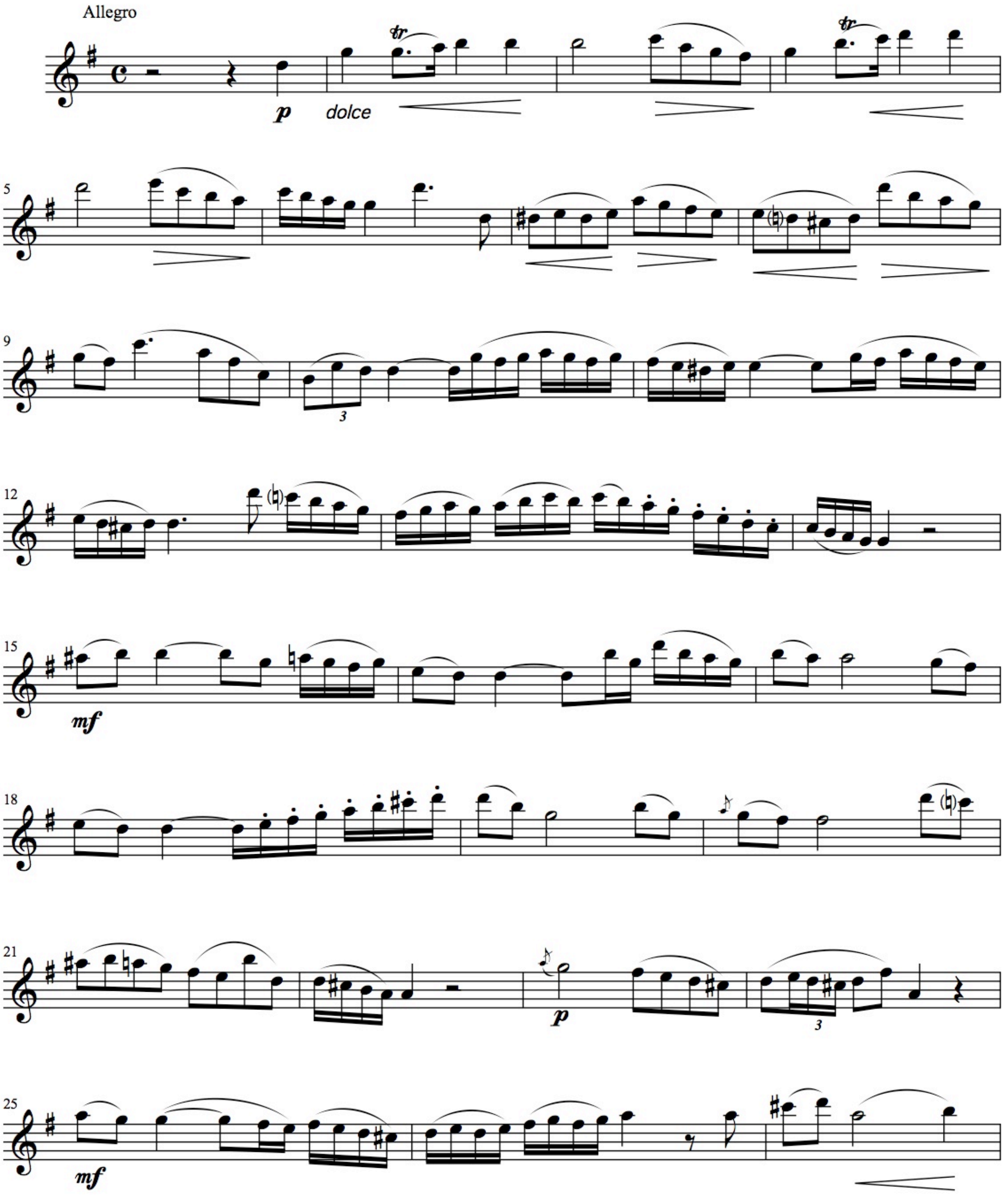

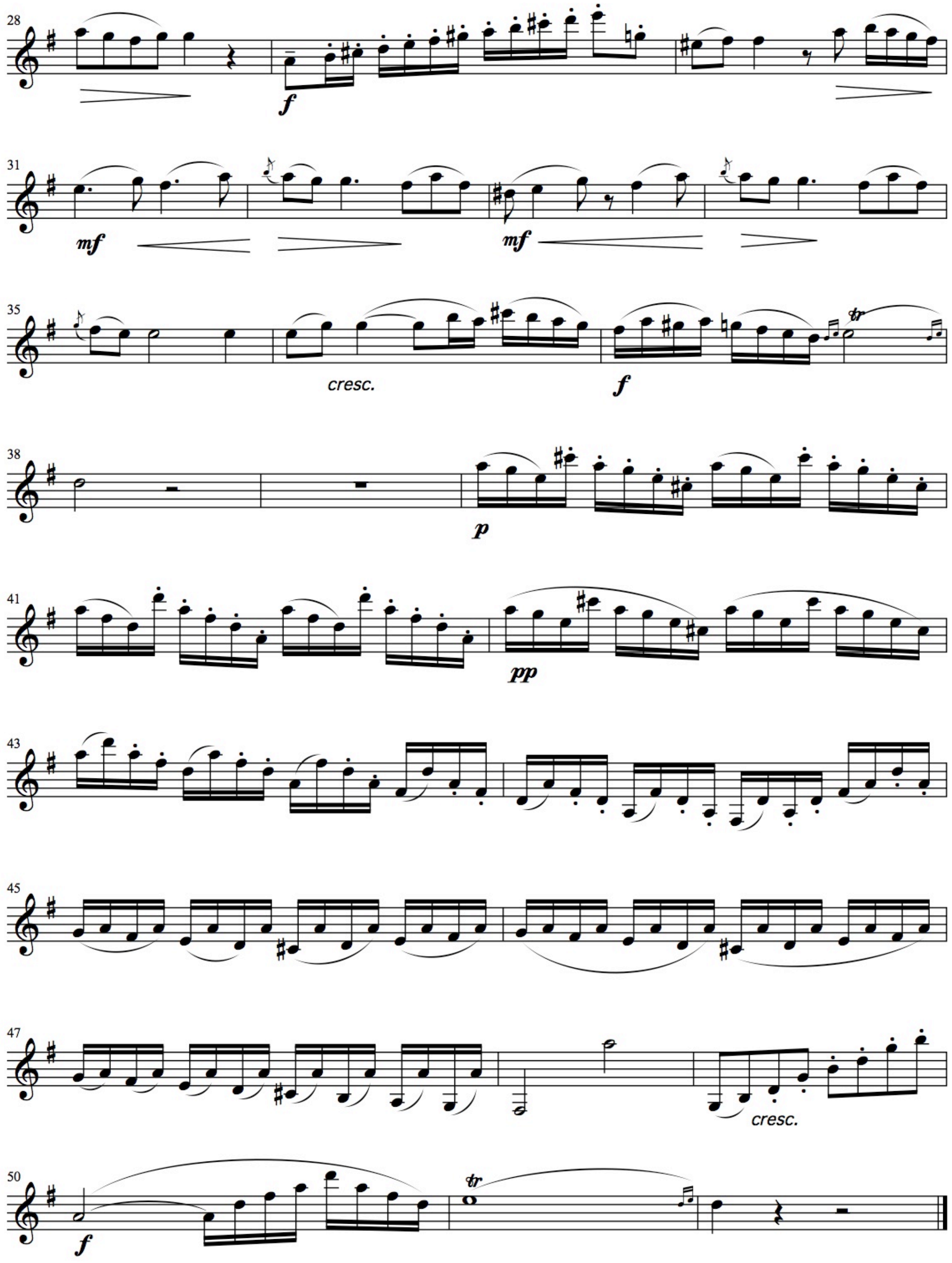
cresc.
cresc.

Supplementary Figure 2: Karl Stamitz, Clarinet Concerto in E-flat Major: I, Exposition
© Friedrich Hofmeister Musikverlag (1956), Leipzig, all rights reserved

Solo-Klarinette in B♭

Konzert in Eb-Dur

(Darmstädter Konzert)

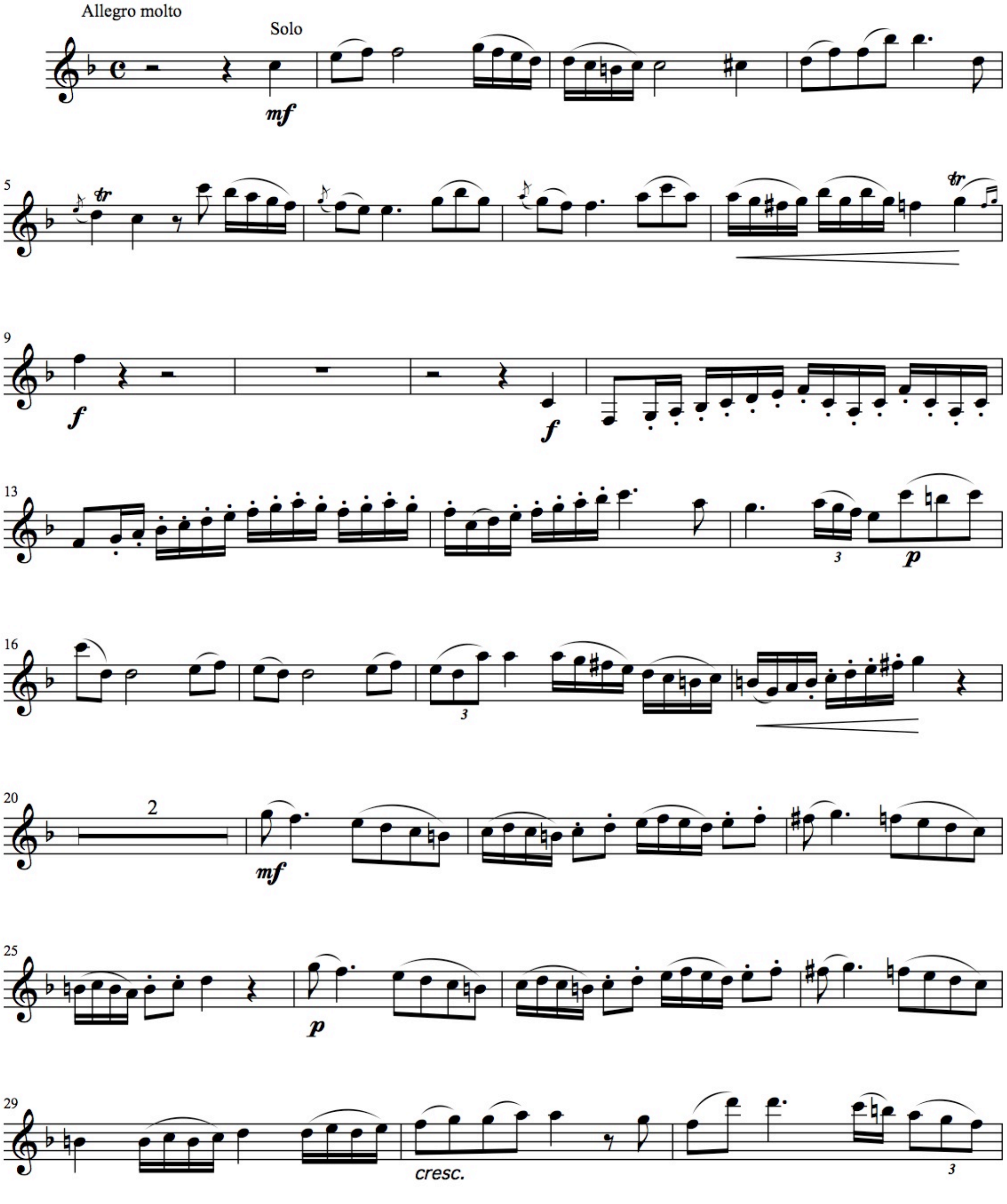

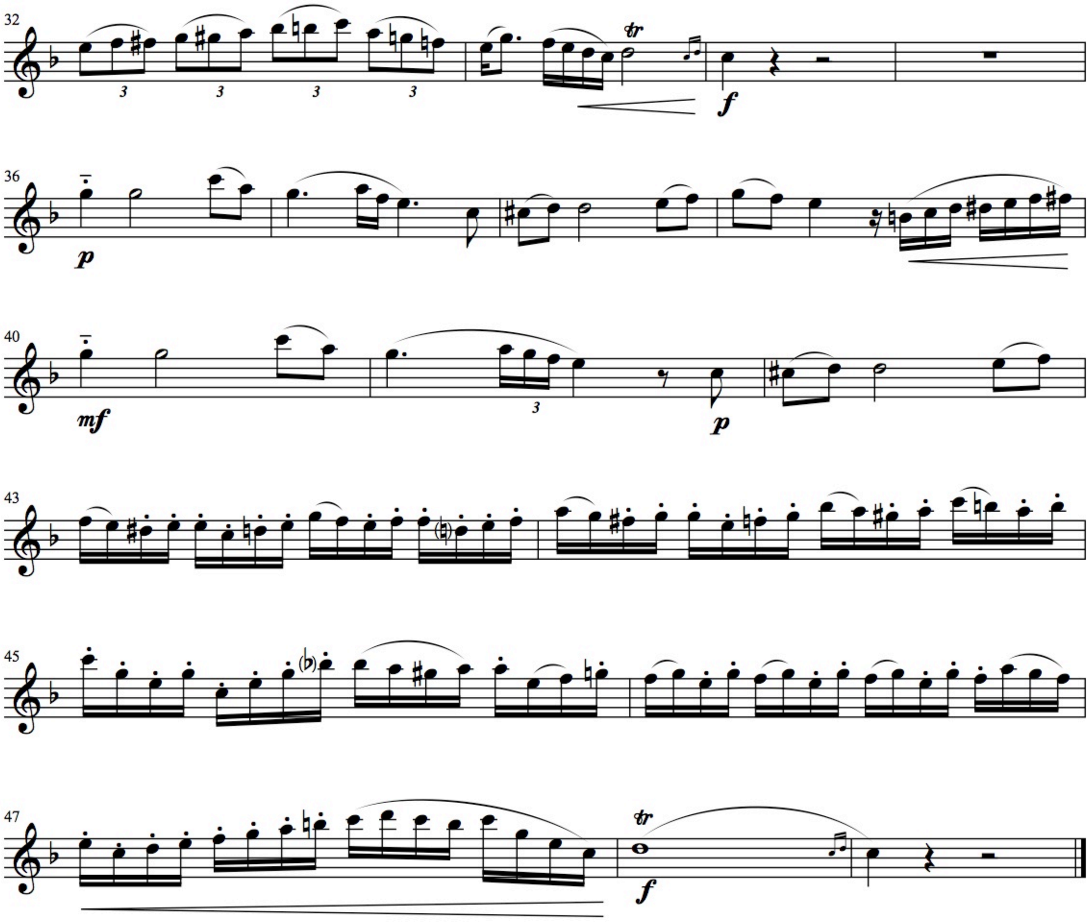
32
tr
f
36
p
40
mf
p
43
45
47
tr
f

Supplementary Figure 3: Jean-Xavier Lefèvre, Progressive Exercise #6
From Jean Xavier Lefèvre's *Méthode de Clarinette (Metodo Per Clarinetto)*
© Casa Ricordi s.r.l. (1939, renewed 1967), Milano, all rights reserved

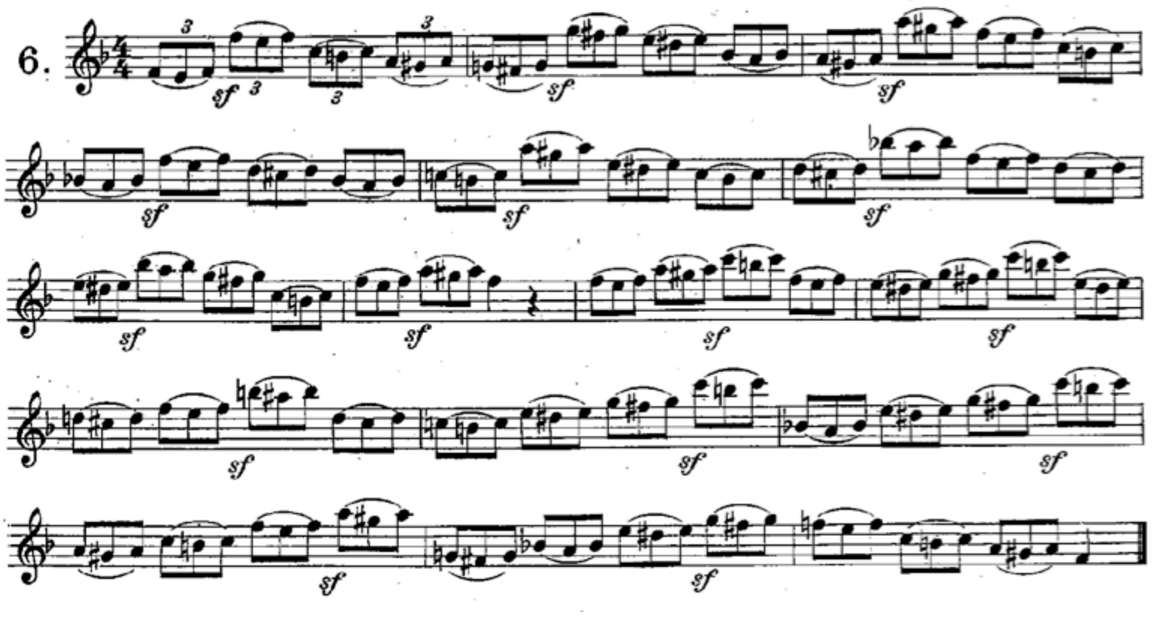

Supplementary Figure 4: Jean-Xavier Lefèvre, Progressive Exercise #19
From Jean Xavier Lefèvre's *Méthode de Clarinette (Metodo Per Clarinetto)*
© Casa Ricordi s.r.l. (1939, renewed 1967), Milano, all rights reserved

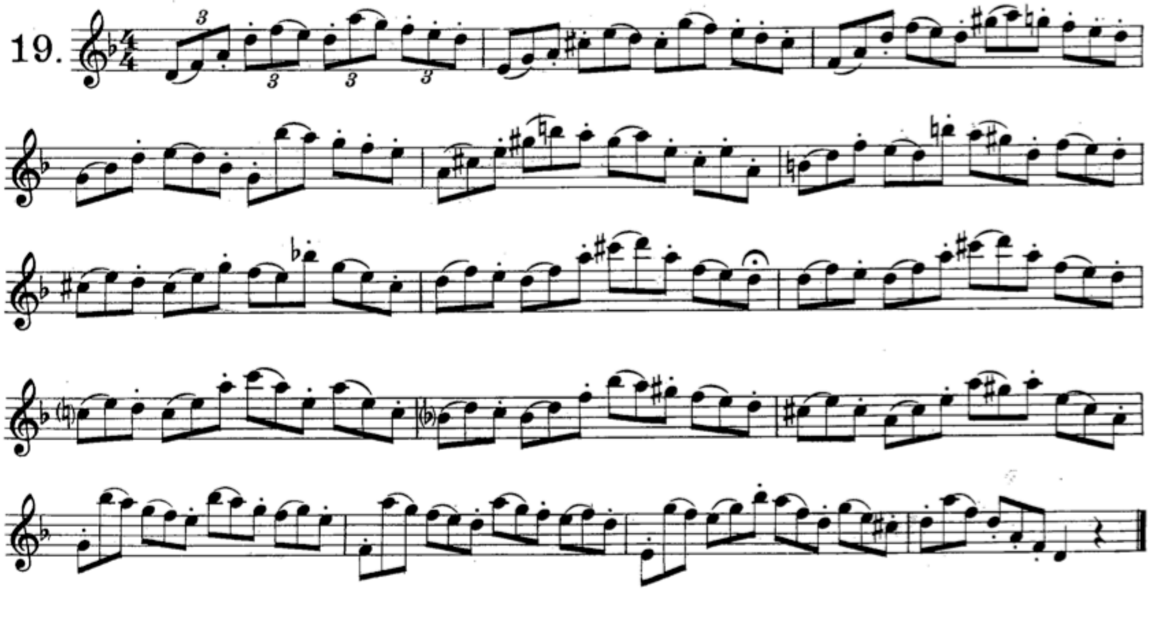

Supplement: Supplementary file 1 [file Image_1.PDF]
